# Athletics and Arteriosclerosis

**Published:** 1904-11

**Authors:** 


### ATHLETICS AND ARTERIOSCLEROSIS.

We are just entering upon the period of the year when, with the opening of the universities, a very large number of young men in this country settle down to the hardest kind of athletic training for the various sports. It seems not inopportune then to review for a moment certain recent expressions of medical opinion with regard to the immediate and remote effects of the severer forms of athletics upon the general health, exercise, and especially those sequelæ that may influence length of life. Not a few of those who took part in the very interesting discussion on arteriosclerosis at the last meeting of the American Medical Association insisted on the fact that the violent forms of exercise most in vogue at universities at the present time are likely to be sources of early arterial degeneration with consequent early loss of vigor and curtailment of life.

As far back almost as medical history runs, it has been conceded that the most harmful factor in breaking down health and in shortening life was severe labor during the period of adolescence and bodily development. This would include especially the years between fifteen and twenty-three, when, after puberty, the body is gradually reaching its acme of development. It is considered almost a truism to say that strenuous physical labor at this time, if carried on to the fullest limit of capacity, is sure to lower resistive vitality and hinder proper development. There are not many who would hesitate to say that to compel growing boys between fifteen and twenty to strain themselves lifting heavy weights, which they are just able to carry, or to work so hard that they sometimes faint because their hearts refuse to send a sufficient amount of blood to the brain, is actually criminal. In what the compulsion to labor so hard differs from athletic exercises which call for just as much exertion and produce as serious effects upon the individual is hard to understand. It is this point particularly that a number of good clinical authorities at Atlantic City insisted on, and suggested that the faculties of our universities should be made to realize.

"A man is as old as his arteries" is a popular truism that remains quite as true for the physician as for the general public. The etiology of premature degeneration of arteries is well-summed up in the expression of the distinguished American clinician who says that it occurs especially in the devotees of the three pagan deities, Vulcan, Venus and Bacchus, that is, it occurs in those who work too hard, in those who suffer from venereal disease rather than from excesses in venery, and in those who indulge too much in spirituous liquors. The relative etiological importance of the causative agent at work is expressed by the order in which they are mentioned above.

There is no single factor in the production of precocious senility due to degeneration of arteries so effective as hard physical labor. The series of statistics presented at the recent meeting of the American Medical Association with regard to the development of arteriosclerosis under forty-five years of age brought out this fact very forcibly. It is no wonder then that there should be agreement among physicians as to the utter inadvisability and almost inevitable harmfulness of the severe physical strain of certain phases of modern athletics.

There is another important element also likely to be harmful in its tendency, to which attention was not directly called although certain of those who took part in the discussion did insist on the necessity for careful dieting and especially on the avoidance of excesses in animal foods, if arteries are to be kept in their normal elastic condition. There is no doubt that an excessive nitrogenous diet adds its quota of irritation to nerves and arteries and leads to unfortunate consequences. The strain put upon the kidneys by such a form of diet is much more severe than with the ordinary mixed diet containing a larger proportion of carbohydrates, which is much more suitable for the adolescent period. There are forms of heart and kidney disease that consequently develop in association with arterial degeneration, and it has never as yet been decided just in which set of organs the pathological condition is primary.

There would seem to be no doubt then that those who apparently are in the best position to judge are agreed that our modern athletics are sure to do much harm unless more carefully regulated. Under these circumstances it might be expected that university faculties would take cognizance of these conclusions and insist on limitation of present-day customs with regard to the severe forms of athletics and the training for them.—*New York Medical News.*

